# To: Neurocritical care management supported by multimodal brain monitoring after acute brain injury

**DOI:** 10.62675/2965-2774.20240276-en

**Published:** 2024-07-02

**Authors:** Josef Finsterer, Fulvio Alexandre Scorza

**Affiliations:** 1 Neurology and Neurophysiology Center Vienna Austria Neurology and Neurophysiology Center - Vienna, Austria.; 2 Universidade Federal de São Paulo Escola Paulista de Medicina Discipline of Neuroscience São Paulo SP Brazil Discipline of Neuroscience, Escola Paulista de Medicina, Universidade Federal de São Paulo - São Paulo (SP), Brazil.

**To the Editor**

We read with interest the article by Monteiro et al. on a retrospective single-center study of the outcomes and mortality of 389 patients with traumatic brain injury (TBI) or subarachnoid bleeding (SAB) depending on the level of neuro-monitoring (standard, advanced) in a neuro-critical care unit (NCCU, Group G1) and a general intensive care unit (ICU) (GICU, Group G2).^( 1 )^ The severity of the disease was assessed at admission to the emergency department using the simplified acute physiology (SAPS) II score.^( 1 )^ Advanced multimodal brain monitoring, including autoregulation and NCCU management, was associated with better outcomes than standard neuromonitoring in the GICU.^( 1 )^ The study is impressive, but some points require discussion.

The major limitation of the study is that factors other than ICU monitoring and ICU type were not adequately included in the evaluation. The outcomes of TBI and SAB depend not only on the type and quality of neuro-monitoring in the ICU but also on several other influencing factors. These include the type and severity of TBI and SAB, the treatment of TBI and SAB, comorbidities, comedication, family history, and genetic background. In addition, for patients with SAB, it must be clarified whether the bleeding is aneurysmal or non-aneurysmal. In the case of an aneurysm, it is important to know whether the aneurysm is coiled or resected. The outcome of SAB may also depend on the initial Hunt–Hess score and whether there is blood inside the ventricles as well as age, comorbidities, the location of the aneurysm, the presence of vasospasms, the presence or absence of strokes, and current medication. For TBI patients, the outcome may depend heavily on whether the patient undergoes surgery. The SAPS II score may not be sufficient to assess the severity of TBI or SAB.

A second limitation is that the number of patients with TBI and SAB in Group 1 and Group 2 was not specified.^( 1 )^ Knowing the proportion of SAB and TBI in Group 1 and Group 2 is crucial because the outcome of both can vary significantly, as can the type of monitoring. Therefore, the ratio of the two in each group can influence the results.

A third limitation is that the Hunt–Hess score was not reported for individuals with SAB.^( 1 )^ Knowledge of the Hunt-Hess score for SAB is crucial because it can assess the severity of SAB more accurately than the Glasgow coma scale (GCS).

A fourth limitation is that GICU patients had a significantly lower mean GCS score and a significantly higher SAPS II score at admission than NCCU patients. Therefore, it cannot be ruled out that the better outcome of NCCU patients was influenced by this initial selection bias and was not related to the type or quality of neuro-monitoring. This possibility should be discussed. There was also a lower GCS score for NCCU patients who received standard monitoring and NCCU patients who received advanced neuromonitoring.

A fifth limitation is that there is no discussion of the side effects of invasive monitoring. The more invasive cerebral monitoring is, the greater the likelihood of side effects, such as infection, dislocation of a probe, the need for reimplantation, or failure of EEG recordings.

In summary, this interesting study has limitations that put the results and their interpretation into perspective. Addressing these issues would strengthen the conclusions and improve the status of the study. Before concluding that advanced neuromonitoring in an NCCU improves outcomes and mortality in SAB and TBI patients, the compared cohorts must be homogenized with respect to initial disease severity and other factors that influence outcomes.
