# Supplementary material for: To: Neurocritical care management supported by multimodal brain monitoring after acute brain injury
Source: Crit Care Sci. 2024 Jul 2;36:e20240276en. doi: 10.62675/2965-2774.20240276-en (PMC11239202; doi:10.62675/2965-2774.20240276-en)
Supplement: Translation

# Translation: Para: Abordagem de cuidados neurocríticos apoiada por monitorização cerebral multimodal após lesão cerebral aguda

**DOI:** 10.62675/2965-2774.20240276-pt

**Authors:** Josef Finsterer, Fulvio Alexandre Scorza

**Affiliations:** 1 Viena Áustria Neurology and Neurophysiology Center - Viena, Áustria.; 2 São Paulo SP Brasil Disciplina de Neurociência, Escola Paulista de Medicina, Universidade Federal de São Paulo - São Paulo (SP), Brasil.

**Ao Editor**

Lemos com interesse o estudo retrospectivo e unicêntrico de Monteiro et al. sobre os desfechos e a mortalidade de 389 pacientes com traumatismo craniencefálico (TCE) ou hemorragia subaracnóidea (HSA) de acordo com o nível de monitoramento cerebral (padrão, avançado) em uma unidade de cuidados neurocríticos (UCN, Grupo 1) e uma unidade de terapia intensiva (UTI) geral (UTI geral, Grupo 2).^( 1 )^A gravidade da doença foi avaliada na admissão ao serviço de emergência utilizando o instrumento *Simplified Acute Physiology Score II* (SAPS II).^( 1 )^O monitoramento cerebral multimodal avançado com autorregulação e abordagem na UCN foi associado a melhores desfechos em relação ao monitoramento cerebral padrão na UTI geral.^( 1 )^O estudo é impressionante, mas alguns pontos precisam ser discutidos.

A maior limitação do estudo é que fatores além do nível de monitoramento e tipo de UTI não foram adequadamente incluídos na avaliação. Os desfechos de TCE e HSA dependem não apenas do tipo e da qualidade do monitoramento cerebral na UTI, mas de diversos outros fatores de influência. Alguns desses fatores incluem o tipo e a gravidade de TCE e HSA, o tratamento para TCE e HSA, as comorbidades, a comedicação, o histórico familiar e a genética. Além disso, em pacientes com HSA, é necessário esclarecer se a hemorragia é aneurismática ou não aneurismática. Em caso de aneurisma, é importante saber se foi embolizado ou ressecado. O desfecho da HSA também pode depender do escore inicial de Hunt-Hess e se há sangue dentro dos ventrículos, bem como da idade, de comorbidades, da localização do aneurisma, da presença de vasoespasmos, da presença ou ausência de acidente vascular cerebral e dos medicamentos em uso. Nos casos de TCE, o desfecho pode depender bastante se o paciente foi submetido à cirurgia ou não. O escore SAPS II pode não ser suficiente para avaliar a gravidade do TCE ou da HSA.

A segunda limitação é que o número de pacientes com TCE e HSA no Grupo 1 e no Grupo 2 não foi especificado.^( 1 )^Saber a proporção de TCE e HSA em cada um desses grupos é crucial, pois o desfecho de cada um pode variar significativamente, assim como o tipo de monitoramento. Portanto, a proporção dos dois em cada grupo pode influenciar os resultados.

A terceira limitação é que o escore de Hunt-Hess não foi relatado para indivíduos com HSA.^( 1 )^Essa informação é crucial, pois avalia a gravidade da HSA de forma mais acurada do que a escala de coma de Glasgow (ECG).

A quarta limitação é que pacientes da UTI geral tiveram pontuação média significativamente menor na ECG e pontuação significativamente maior no SAPS II na admissão do que pacientes da UCN. Por isso, não se pode descartar que a obtenção de melhores desfechos em pacientes da UCN foi influenciada por esse viés de seleção inicial e não estava relacionada ao tipo e nem à qualidade do monitoramento cerebral. Essa possibilidade deve ser discutida. Além disso, pacientes da UCN que receberam monitoramento padronizado e pacientes de UCN que receberem monitoramento avançado também apresentaram pontuação menor na ECG.

A quinta limitação é que não há discussão sobre os efeitos colaterais do monitoramento invasivo. Quanto mais invasivo for o monitoramento cerebral, maior será a probabilidade de efeitos colaterais, como infecção, deslocamento de sonda, necessidade de reimplante ou falha nos registros de eletroencefalograma.

Em resumo, esse estudo tem limitações que colocam os resultados e suas interpretações em questão. Abordar esses problemas reforçaria as conclusões e melhoraria a qualidade do estudo. Antes de concluir que o monitoramento cerebral avançado na UCN melhora os desfechos e a mortalidade em pacientes com TCE e HSA, as coortes comparadas devem ser homogeneizadas em relação à gravidade inicial da doença e a outros fatores que influenciam os desfechos.
